# Exploring the metabolic and cuticular mechanisms of increased pyrethroid resistance in *Anopheles gambiae* S.l populations from Ghana

**DOI:** 10.1038/s41598-025-03066-9

**Published:** 2025-05-29

**Authors:** Miriam DedeAma Dortey, Anisa Abdulai, Isaac Kwame Sraku, Judith Dzifa Azumah, Isaac Anim-Baidoo, Yaw Asare Afrane

**Affiliations:** 1 Centre for Vector-Borne Disease Research, Department of Medical Microbiology, University of Ghana Medical School, Accra, Ghana; 2 Department of Medical Laboratory Sciences, School of Biomedical and Allied Health Sciences, College of Health Sciences, University of Ghana, Accra, Ghana

**Keywords:** *Anopheles gambiae* S.l, Insecticide resistance, Metabolic, Cuticular, CYP450s, Ghana, Entomology, Medical research

## Abstract

Increasing insecticide resistance in malaria vectors threatens the efficacy of current control tools, however knowledge of metabolic and cuticular mechanisms is widely lacking in Ghana. We examined the metabolic and cuticular resistance mechanisms in *Anopheles gambiae* mosquitoes from coastal and sahel zones of Ghana. WHO susceptibility tests and synergist assays were performed on F0 field collected *An. gambiae* s.l. Gene expression profiles of eight key metabolic and cuticular genes were determined using qRT-PCR. Moderate to high pyrethroid resistance (< 70%) were observed across all the sites. Piperonyl butoxide significantly increased susceptibility to pyrethroids across all sites and insecticides, implicating P450s. Gene expression analysis revealed overexpression of metabolic and cuticular resistance genes in field *An. gambiae* populations compared to the susceptible Kisumu strain. *CYP6M2* and *CYP6P3* were the most overexpressed metabolic genes in pyrethroid-resistant mosquitoes, compared to the pyrethroid susceptible mosquitoes in the coastal (FC: 122.28 and 231.86, *p* < 0.05) and sahel (FC: 344.955 and 716.37, *p* < 0.001) zones respectively. *CYP4G16* (previously associated with cuticular resistance) was significantly overexpressed in only resistant mosquitoes (FC: 3.32–30.12, *p* < 0.05). Overexpression of metabolic and cuticular resistance genes in local malaria vectors highlights the need to intensify insecticide resistance management strategies to control malaria in Ghana.

**Supplementary Information:**

The online version contains supplementary material available at 10.1038/s41598-025-03066-9.

## Introduction

Vector control remains the mainstay of malaria control and elimination efforts in Sub-Saharan Africa (SSA)^1^. Over the last decade, the widespread deployment of insecticide-based vector control tools such as long-lasting insecticidal nets (LLINs) and indoor residual spraying (IRS) has significantly reduced malaria transmission in SSA^2,3^. However, increasing insecticide resistance in malaria vectors poses a significant threat to malaria control efforts^4–6^. High insecticide resistance has been reported in many countries in SSA, with varying resistance profiles often mediated by the widespread use of insecticides in agriculture and public^7–9^. Several studies in Ghana have reported increasing resistance to multiple insecticide classes, including pyrethroids, organophosphates, and carbamates in local malaria vector populations^10,11^.

Target site resistance and metabolic resistance are the two main mechanisms of insecticide resistance in malaria vectors^12^. However, other resistance mechanisms, namely cuticular and behavioral resistance have been involved in the development of insecticide resistance in malaria vectors^13,14^. Knockdown resistance (KDR) mutations in the target site for insecticides such as L1014 F, L1014S and N1570Y have been involved in pyrethroid resistance in African malaria vectors^10,15^. Knockdown resistance mutation, L1014 F has been found to be nearing fixation in local malaria vectors in Ghana^10,11^, signifying that other resistance mechanisms may be involved in the development of insecticide resistance.

Major metabolic enzymes such as cytochrome P450 s (CYP450s) monoxygenases, glutathione-s-transferases and esterases have been implicated in insecticide resistance in malaria vectors^12,16,17^. Overexpression of these metabolic enzymes especially CYP450 s has been linked to pyrethroid resistance in malaria vectors^18,19^. Overexpression of *CYP6P3*,* CYP6P4*,* CYP6Z1*,* CYP6Z2*,* CYP6M2*, and *CYP9K1* has been linked to pyrethroid resistance in *An. gambiae*^12,20^. A study by Dadzie et al.^21^ showed that the use of Piperonyl butoxide (PBO) significantly enhanced the susceptibility of *An. gambiae* s.l mosquitoes in Ghana. Piperonyl butoxide is a synergist that inhibits metabolic enzyme, cytochrome P450 s (CYP450s) monoxygenases. This suggests that metabolic resistance may be involved in the resistance observe in local malaria vectors in Ghana.

Very little attention has been given to cuticular resistance in malaria vectors in SSA. Cuticular resistance involves modifications in the mosquito’s exoskeleton, making it more difficult for insecticides to penetrate and reach target sites^22^. A study by Wood et al.^23^ found that significantly greater cuticle thickness in permethrin resistant mosquitoes compared to susceptible ones. A study by Yahouedo et al.^24^ also found some cuticle genes, *CPLCG3*, *CPR124* and *CPR129* to be overexpressed in resistant *An. gambiae* mosquitoes and possibily involed in cuticular resistance. The overexpression of the cytochrome P450, *CYP4G16* has been associated with cuticle reinforcement in resistance^24^.

**Fig. 1:**
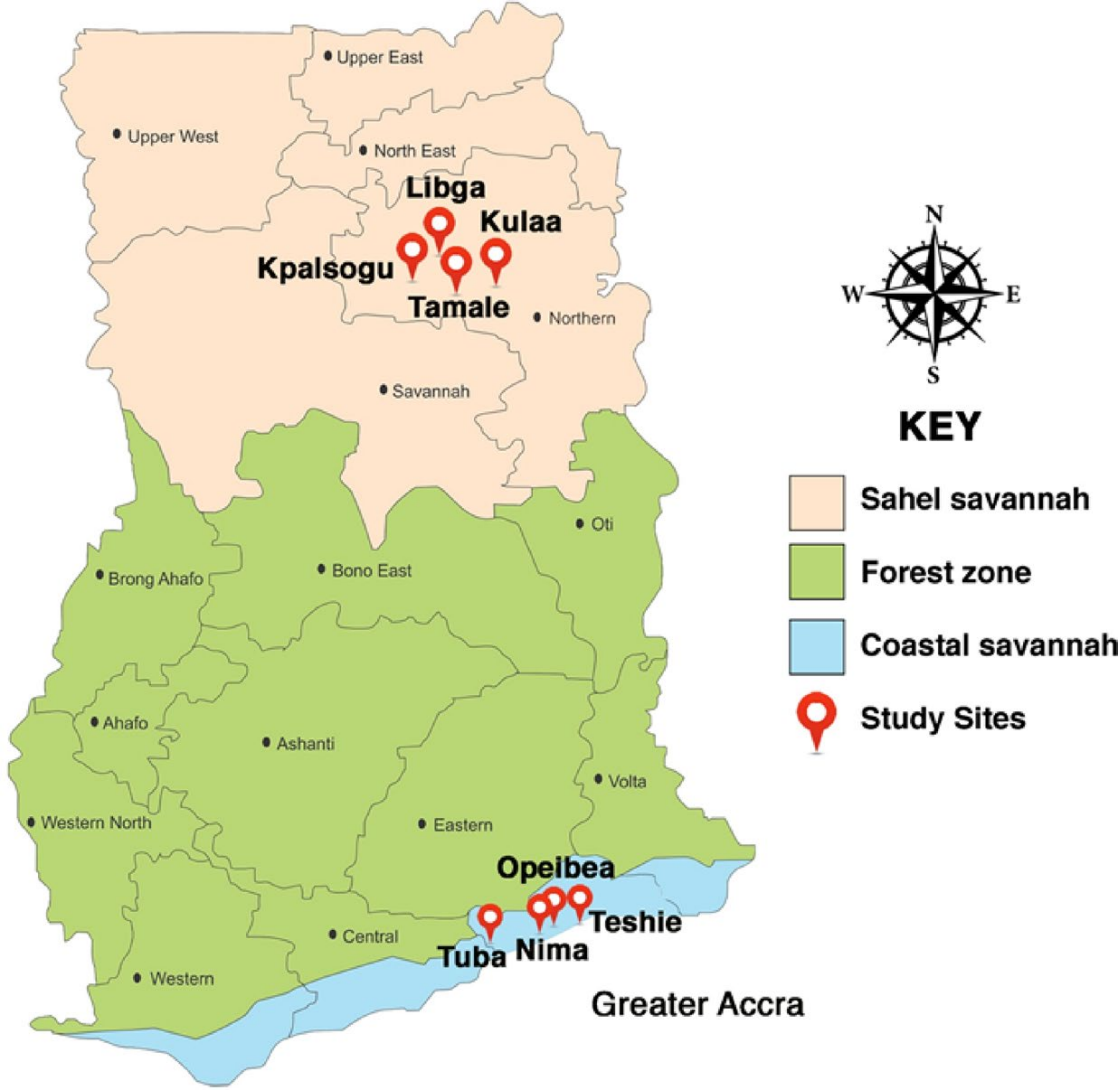
Map of Ghana showing the study sites.

Understanding the role of metabolic and cuticular resistance in local malaria vectors in Ghana will help in enhancing the effectiveness of current vector control tools for malaria elimination. Currently, there is paucity of data on the specific metabolic and cuticular genes that may be involved in insecticide resistance in malaria vectors in Ghana. The coastal and sahel zones of Ghana exhibits significant variations in climate, agricultural practices, insecticide use and malaria transmission dynamics, factors that could influence the selection pressure and development of resistance in local mosquito populations^25^. Therefore, a comparative study of insecticide resistance mechanisms in *An. gambiae* s.l. populations from these regions is essential to identify localized resistance profiles and inform tailored control strategies. This study aims to investigate the phenotypic, metabolic, and cuticular mechanisms of insecticide resistance in *An. gambiae* s.l. in coastal and sahel ecological zones of Ghana. (Fig. 1)

## Results

### Phenotypic resistance in *Anopheles gambiae* S.l

**Fig. 2:**
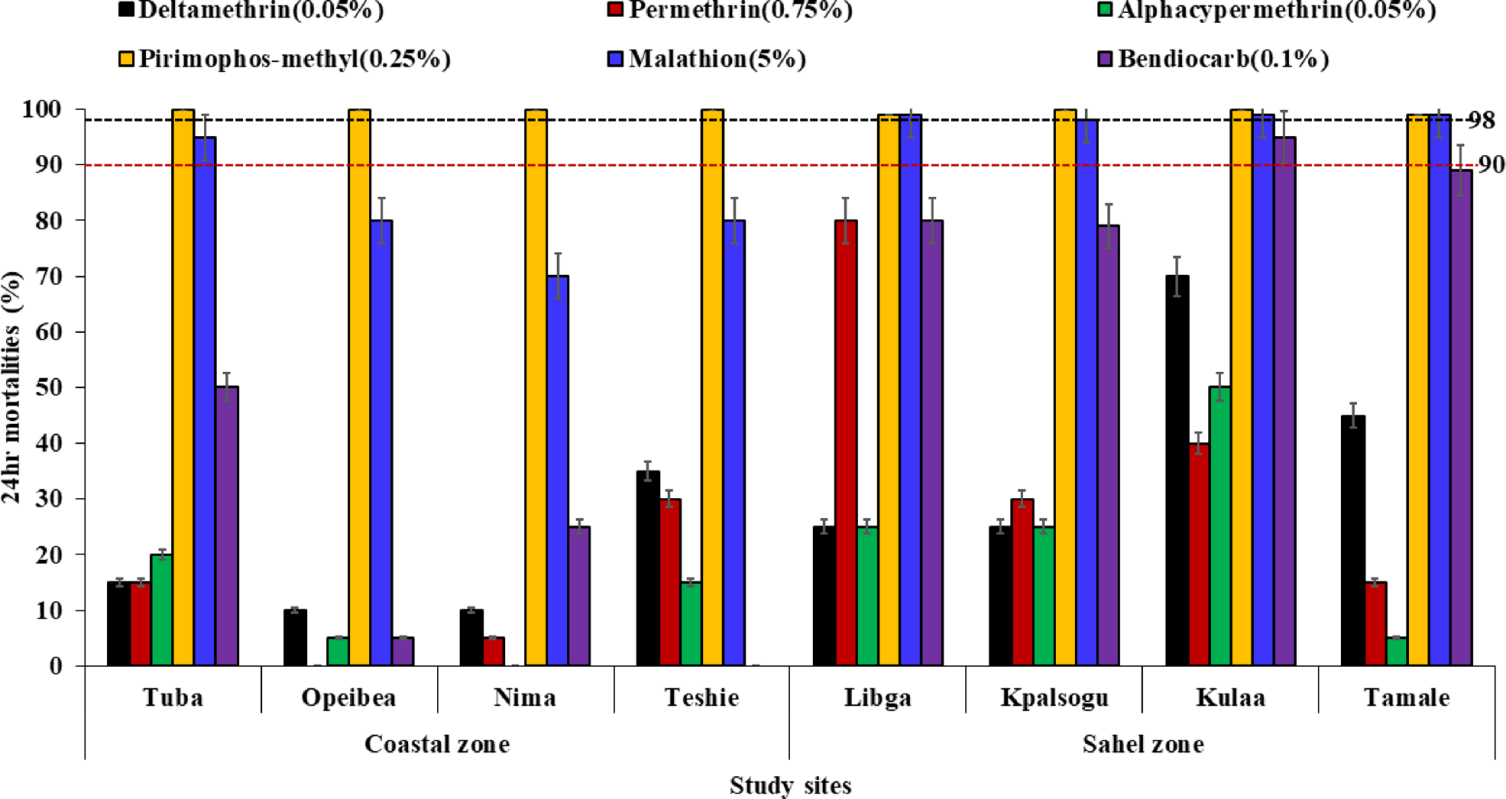
24-hr mortalities of *Anopheles* mosquitoes with exposure to insecticides, permethrin, deltamethrin, alphacypermethrin, malathion, pirimiphos-methyl and bendiocarb. Error bars represent the 95% confidence interval of the mean.

*Anopheles gambiae* from all study sites in the coastal and sahel zones showed high to moderate resistance to all the pyrethroids insecticides. Resistance to permethrin was also detected in each site: [Coastal sites, Tuba (15%), Opeibea (0), Nima (5%) and Teshie (30%) and Sahel sites, Libga (80%), Kpalsogu (30%), Kulaa (40%) and Tamale (15%) with significant variations in mortalities across the sites (χ2 = 183.057, *df* = 7, *p* < 0.001). Low mortality rates to deltamethrin [Coastal sites (10–35%) and Sahel sites (25–70%)] were also observed in all the sites (χ2 = 112.672, *df* = 7, *p* < 0.001). Mortality rates to alphacypermethrin were significantly lower in the *Anopheles gambiae* populations from Nima (0%) compared to all the other sites ((χ2 = 96.868, *df* = 7, *p* < 0.001) (Fig. 2). In the coastal zone, all mosquitoes from Opeibea, Nima and Teshie were resistant to malathion (70–80%) except in Tuba, which showed suspected resistance (95%). However, in the Sahel savannah sites, *An. gambiae* were all susceptible to malathion (> 98%). Similarly, *Anopheles* mosquitoes were susceptible to pirimiphos methyl in all the sites with 100% mortality rates observed in all sites except Libga (99%) and Tamale (99%). Mosquitoes were resistant to bendiocarb in all the sites (0 to 89%) except in Kulaa (95%) and Tamale (90%) (Fig. 2). Chi-square analysis showed significant differences in mortalities to the pyrethroids, bendiocarb and malathion between ecological zones and study sites (*p* < 0.05).

### Synergist assays

**Fig. 3:**
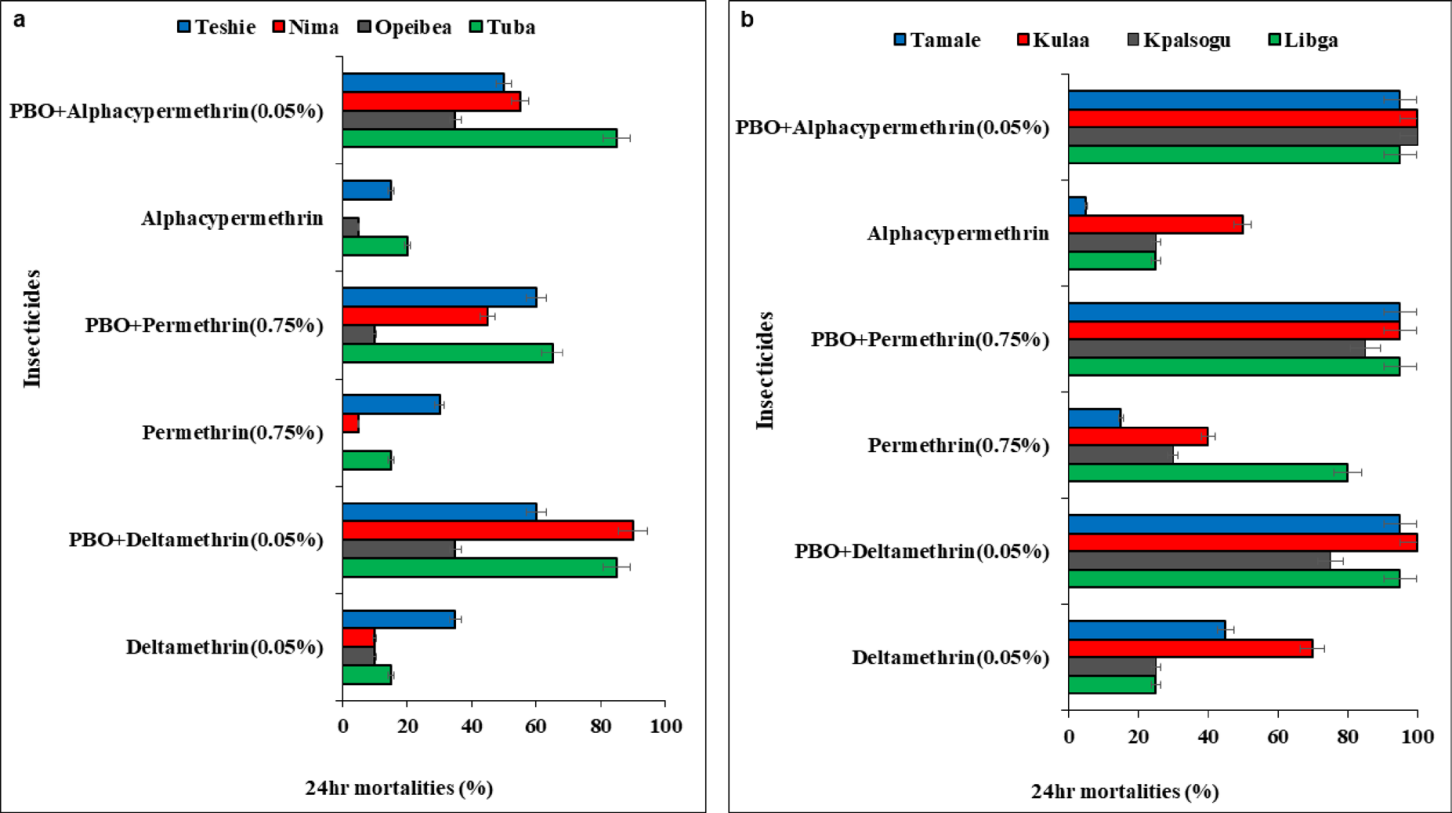
Synergistic effects of PBO on the insecticide susceptibility status of *Anopheles* gambiae s.l populations from the study sites. **a**–**b** the 24-h mortalities of *Anopheles* mosquitoes from Coastal zone (**a**) and Sahel zone (**b**) respectively.

Piperonyl butoxide (PBO) increased the susceptibility of *An. gambiae* to pyrethroids across all the sites and insecticides. In the coastal zone, pre-exposure of *An. gambiae* to PBO increased susceptibility to deltamethrin [Tuba (15–85%), Opeibea (0 to 10%), Nima (10–90%) and Teshie (35–60%)], permethrin [Tuba (15–65%), Opeibea (10 to 35%), Nima (5 to 45%) and Teshie (30 to 60%) and alphacypermethrin [Tuba (20–85%), Opeibea (5 to 35%), Nima (15 to 50%) and Teshie (0 to 55%)]. Despite the increase in mortality, there was no complete restoration of susceptibility observed for *An. gambiae* in the coastal zone (Fig. 3a).

For *An. gambiae* s.l mosquitoes from the Sahel zone, pre-exposure to PBO led to partial restoration of susceptibility to deltamethrin in all the sites except for Kulaa (70 to 100) where there was complete restoration of susceptibility. Increased mortality after pre-exposure to permethrin was observed in all the Sahel sites [Libga (80–95%), Kpalsogu (30% to 85), Kulaa (40–95%) and Tamale (15–95%). There was complete restoration of susceptibility to alphacypermethrin after pre-exposure to PBO in Kpalsogu (25–100%) and Kulaa (50–100%) but partial restoration of susceptibility in the other Sahel sites (Fig. 3b). For all Synergist-insecticide combinations, there was a significant difference in mortality between study sites (*p* < 0.05).

### Species distribution of *Anopheles* gambiae S.l mosquitoes from the study sites

A subsample of 400 *An. gambiae* s.l. from all the study sites was randomly selected and used to discriminate the sibling species. *Anopheles coluzzii* was the most abundant species (41.75%, 167/400) followed by *Anopheles arabiensis* (28.75%, 115/400), *Anopheles gambiae s.s.* (27.75%, 111/400) and the least were the hybrids of *An. coluzzii* and *An. gambiae s.s.* (1.7%, 7/400). *An. arabiensis* was only detected in the Sahel zone in significant numbers especially in Tamale (84%, 42/50) and Kulaa (82%, 41/50). Table 1 shows the species discrimination of *Anopheles gambiae* s.l. across the study sites.

**Table 1:** Species discrimination *of Anopheles gambiae* S.l. *In the study sites*.

Site	Ecological zone	Species, *N* (%)				
		An. coluzzii	An. gambiae s.s.	An. arabiensis	Hybrids	Total
Kpalsogu	Sahel zone	24 (48)	9 (18)	17 (34)	0	50 (100)
Tamale		8 (16)	0	42 (84)	0	50 (100)
Libga		26 (52)	9 (18)	15 (30)	0	50 (100)
Kulaa		9 (18)	0	41 (82)	0	50 (100)
Opeibea	Coastal zone	8 (16)	42 (84)	0	0	50 (100)
Teshie		29 (58)	19 (38)	0	2 (4)	50 (100)
Tuba		31 (62)	15 (30)	0	4 (8)	50 (100)
Nima		32 (64)	17 (34)	0	1 (2)	50 (100)
Total		167 (41.75)	111 (27.75)	115 (28.75)	7 (1.75)	400 (100)

N: frequency, %: percentage.

### Gene expression profiles of *An. gambiae* S.l. Across ecological zones

**Fig. 4:**
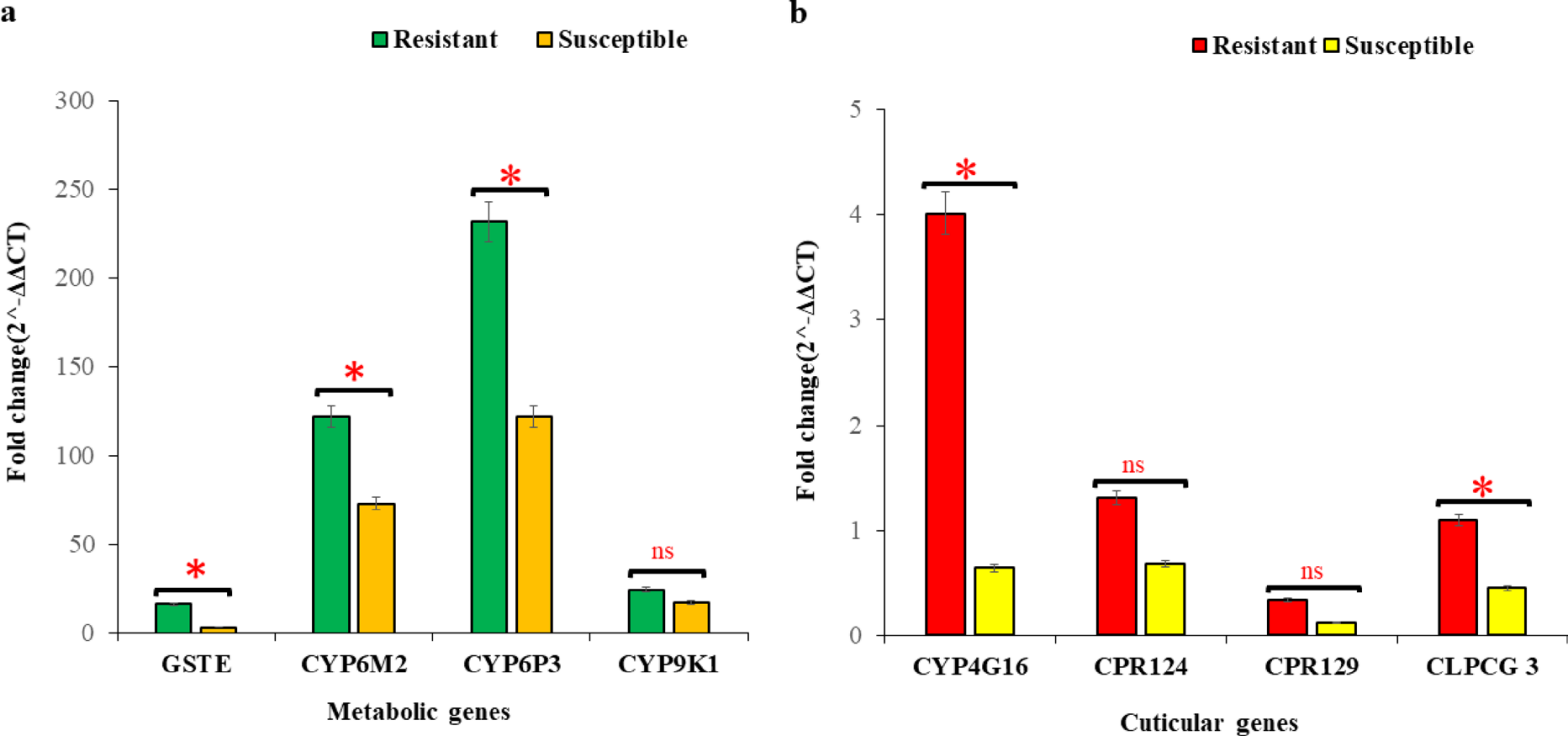
Fold expression of metabolic and cuticular genes in *An. gambiae* populations from the Coastal zone of Ghana. (**a** – **b**) shows the gene expression profiles of metabolic genes (**a**) and cuticular genes (**b**) respectively. Error bars represent the 95% confidence interval of the mean. * *P* < 0.05, ns = not significant.

Two hundred and forty (240) *An. gambiae* s.l. mosquitoes were tested for the expression of cytochrome P450 genes (*CYP6P3*,* CYP6M2*,* CYP9K1* and *CYP4G16*), one GST gene (*GSTE2*) and three cuticular genes (*CLPCG3*,* CPR124*,* CPR129*) relative to the housekeeping gene RPS7 and compared to Kisumu. In the coastal zone, significantly higher Fold Changes (FC) of the *CYP6M2*,* CYP6P3*, *GSTE2*, *CYP416* and *CLPCG3* were observed in the pyrethroid resistant mosquitoes compared to susceptible mosquitoes (Fig. 4). The highest overexpression in the coastal zone was observed for *CYP6P3* and *CYP6M2* with their resistant group having an average fold change of 231.86 and 122.23 respectively. There were no significant differences in the fold change of *CYP9K1*, *CPR124* and *CPR129* in the resistant and susceptible mosquitoes from the coastal zone (*P* > 0.05) (Fig. 4).

**Fig. 5:**
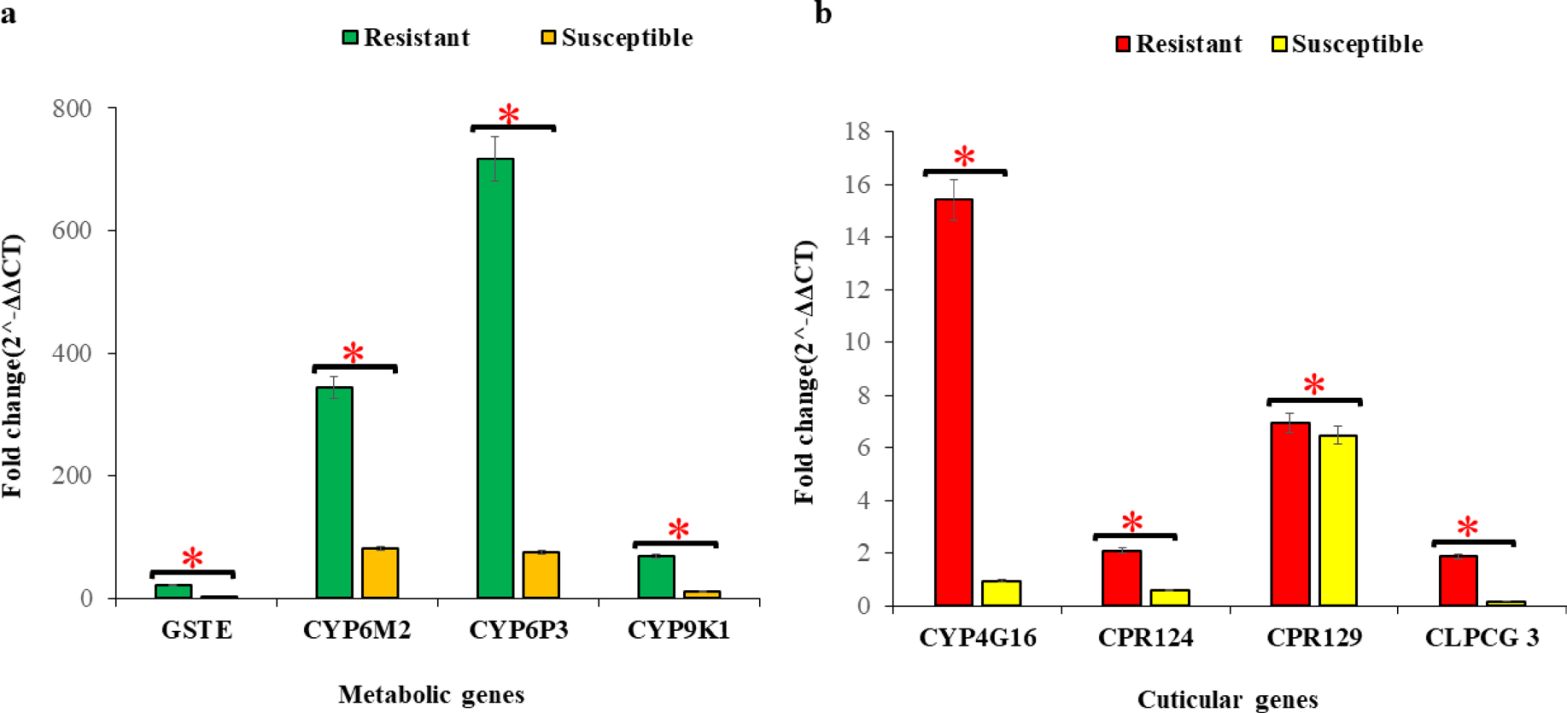
Fold expression of metabolic and cuticular genes in *An. gambiae* populations from the Sahel zone of Ghana. (**a** – **b**) shows the gene expression profiles of metabolic genes (**a**) and cuticular genes (**b**) respectively. Error bars represent the 95% confidence interval of the mean. * *P* < 0.05, ns = not significant.

Gene expression profiles of *An. gambiae* s.l mosquitoes from the Sahel zone of Ghana showed significant differences in fold changes between phenotypically resistant and susceptible for all the genes tested (*P* < 0.05). Like the coastal zone, the highest overexpression was observed in *CYP6P3* (fold change: 716.37) and *CYP6M2* (fold change: 344.96) in the resistant group of *An. gambiae* s.l. mosquitoes (Fig. 5).

### Gene expression profiles across the different study sites

Significant gene expression levels between the Coastal zone and Sahel zone were only observed in the GST gene, *GSTE2* (t (70) = 2.007, *P* = 0.048) and cuticular gene, *CPR129* (Mann–Whitney *Z* = −4.198, *P* < 0.001). Differential expression levels of metabolic and cuticular genes were observed across all the sites. Metabolic genes *CYP6M2*, *CYP6P3* and *CYP6P3* were significantly overexpressed in mosquitoes from all the sites compared to the susceptible Kisumu strain (*P* < 0.05) (Table 1). The metabolic gene *CYP4G16*, which is also involved in cuticular resistance was only found to be significantly overexpressed in only resistant *An. gambiae* mosquitoes from Tuba, Teshie, Libga, Kulaa and Tamale (FC: 3.32–30.12, *P* < 0.05) (Table 2). More information on the fold changes, means and standard deviations of the CT-values compared to kisimu strain are found in Supplementary Data S1.

**Table 2:** Associations between fold changes of metabolic and cuticular genes in pyrethroid resistant and susceptible mosquitoes compared to Kisumu strain.

Study Sites	Phenotype	Fold Change of Metabolic Genes				Fold Change of Cuticular Genes			
		GSTE2	CYP6M2	CYP6P3	CYPK1	CYP4G16	CPR124	CPR129	CLPCG3
Tuba	Resistant	6.07	51.60^**^	91.15^*^	10.14^**^	7.25^*^	0.21	0.10^***^	1.99
	Susceptible	2.17	34.60^*^	39.92	2.32	0.36	0.12^*^	0.28^***^	0.08
Opeibea	Resistance	16.07^*^	339.83^**^	525.93^*^	49.64^***^	1.75	1.64	0.02^***^	0.07^*^
	Susceptible	5.42	183.51^**^	323.95^*^	47.79^***^	0.96	0.27	0.01^***^	0.89
Nima	Resistant	36.21^*^	23.87^*^	115.80^*^	11.12^*^	1.13	2.76	0.25^**^	1.96
	Susceptible	1.62	5.67	68.25^*^	7.50^*^	0.71	1.75	0.41	0.52
Teshie	Resistant	6.84^*^	73.61^*^	194.57^*^	27.79^**^	5.91^*^	0.63	0.09^**^	0.38
	Susceptible	4.49	68.86^**^	56.47^*^	11.20^**^	0.53	0.56	0.67^**^	0.31
Kpalsogu	Resistant	57.70^*^	28.41^*^	200.54^*^	178.58^**^	2.58	5.08	5.24	3.01
	Susceptible	0.12	22.00^*^	52.52*	0.15^*^	0.78	0.37	5.44	0.16
Libga	Resistant	23.68	805.00^**^	1956.88^***^	63.27^*^	30.12^**^	1.51	4.53	2.26
	Susceptible	1.57	68.25^**^	133.59^*^	23.15^**^	2.77	1.34	14.67	0.11
Kulaa	Resistant	0.72	344.71^**^	425.42^*^	21.73^**^	25.68^*^	1.62	9.13	1.82
	Susceptible	0.06	115.25^**^	76.14^*^	13.74^**^	0.15	0.51	3.30	0.29
Tamale	Resistant	1.68	201.70^**^	282.64^*^	15.66^**^	3.32^*^	0.19^*^	8.87^*^	0.44^*^
	Susceptible	0.30	121.10^**^	39.65^*^	6.77^*^	0.04	0.13	2.56	0.08

Associations between the fold changes of genes in phenotypically resistant mosquitoes and susceptible mosquitoes compared to Kisumu. ^*^ shows *P* < 0.05; ^**^ *P* < 0.01; ^***^ *P* < 0.001.

## Discussion

Understanding the role of metabolic and cuticular resistance in local malaria vectors is important to help in current insecticide resistance management efforts and malaria elimination in Ghana. This study investigated the phenotypic, metabolic and cuticular mechanisms of resistance in *Anopheles gambiae* s.l. across the coastal and sahel ecological zones of Ghana. Mosquitoes were resistant to all tested pyrethroids, deltamethrin, permethrin and alphacypermethrin while remaining susceptible to pirimiphos-methyl across all study sites. The metabolic genes *CYP6P3* and *CYP6M2* were highly expressed in both the Coastal and Sahel zones, while cuticular gene expression varied by region, with *CYP4G16* most expressed in these two zones. Pre-exposure to piperonyl butoxide (PBO) increased mosquito mortality to pyrethroids across all sites. Additionally, pyrethroid resistant mosquitoes exhibited higher levels of both metabolic and cuticular resistance genes compared susceptible mosquitoes.

The expressions of key metabolic genes (*CYP9K1*, *CYP6M2*, *CYP6P3*, and *GSTE2*) were found to be upregulated in field *An. gambiae s.s.* compared to the susceptible colony, Kisimu strain. Significant differential expression of these metabolic genes observed between pyrethroid resistant and susceptible mosquitoes suggests that these genes may be involved in pyrethroid resistance in *An. gambiae* populations in Ghana. This may have serious implications on the effectiveness of pyrethroid based vector control interventions such as LLINs across Ghana. *CYP6P3* and *CYP6M2* were found to be overexpressed at a significantly higher fold change compared to *GSTE2* and *CYP9K1* in both resistant and susceptible *An. gambiae* populations relative to the susceptible Kisumu strain across the coastal and sahel zones of Ghana. This findings is in line with literature as *CYP6P3* and *CYP6M2* are considered as the main pyrethroid metabolizing enzymes in several *An. gambiae populations* in West Africa^26–28^. Another study by Adolfi et al.^29^ found *CYP6P3* overexpression to confer resistance to both pyrethroids and carbamates. Our findings suggests that these two genes, *CYP6P3* and *CYP6M2* are greatly involved in metabolic resistance mechanisms of local malaria vectors in Ghana. However, higher expression of *CYP9K1* and *GSTE2* was observed in the resistant *An. gambiae* mosquitoes compared to the susceptible, showing that these genes may also be playing a role in resistance. *CYP9K1* has been found in pyrethroid resistant mosquitoes and have been found to metabolize pyrethroids^20,30^. The glutathione-S-transferase *GSTE2* has also been associated with DDT and pyrethroid resistance^31,32^. Given the prominent role of mixed-function oxidases (MFOs) in pyrethroid resistance and evidence of increased insecticide susceptibility with insecticide-PBO combinations, it is important for the Ghana National Malaria Elimination Program (NMEP) to deploy dual active ingredient LLINs to help in the management of insecticide resistance in local malaria vectors.

Higher expression of cuticular genes *CYP4G16*, *CPR124*, *CPR129*, and *CPLCG3* was observed in resistant *An. gambiae* s.l mosquitoes compared susceptible mosquitoes, suggesting that cuticular mechanisms may also be involved in the observed resistance. Similarly, over-expression of *CPLCG3* and *CPR129* have been found to be associated with insecticide resistance through cuticle remodeling, affecting insecticide penetration rates *in An. coluzzii*^24^. The cytochrome P450, *CYP4G16*, consistently over-expressed in pyrethroid-resistant *Anopheles* mosquitoes has been recently found to be involved in cuticular hydrocarbon production and cuticle reinforcement in resistance in *Anopheles gambiae*^22,24^.

The insecticide resistance profile of *An. gambiae* s.l mosquitoes varied across all the study sites. Resistance to pyrethroids was widespread across all the sites. This suggests that mosquitoes from both regions are likely exposed to similar selection pressures, potentially due to the widespread use of pyrethroids in public health and agriculture. Previous studies in Ghana have also documented high levels of pyrethroid resistance in *Anopheles gambiae* s.l^11,33^, correlating this resistance with reduced efficacy of pyrethroid-based long-lasting insecticidal nets (LLINs) as pyrethroids are the primary active ingredient in aerosol sprays, mosquito coils, and repellents, as well as LLINs^34^.

Mosquitoes were found to be fully susceptible to the organophosphate, pirimiphos-methyl in all the sites. This may be linked to the shift from pirimiphos-methyl to clothianidin for Indoor Residual Spraying (IRS) in the Sahel savannah zone since 2021^35^. This change may have contributed to the restoration of susceptibility to pirimiphos-methyl in the region. In the coastal savannah zone, the absence of IRS activities may also be contributing to the observed susceptibility of vector species in this study. A study in Ghana by Baffour-Awuah et al.^34^ also reported the susceptibility of *Anopheles gambiae* s.l populations to pirimiphos-methyl, suggesting that this insecticide remains effective for malaria control interventions across these sites.

This widespread pyrethroid resistance has serious implications on the efficacy of current pyrethroid based tools such as LLINs in Ghana. The ability of the synergist PBO to restore full or partial susceptibility to permethrin, deltamethrin and alphacypermethrin suggests that mixed-function oxidases (MFOs) contribute to the observed resistance. Similar findings have previously been observed in Ghana and west Africa^21,36^. This highlights the incorporation of the synergist, PBO, into pyrethroid-based vector control tools to increase the susceptibility of local malaria vectors to pyrethroids, especially in high pyrethroid sites.

*Anopheles coluzzii* was the most predominant species identified in all the study sites. *Anopheles coluzzii* prefers permanent larval habitats such as irrigated fields and breeds year-round^37^, which likely explains its dominance in the study sites as also reported in Ghana by Chabi et al.^38^. *Anopheles arabiensis* was exclusively found in the sahel savannah zone, consistent with findings from other studies in Ghana that showed its adaptation to arid environments^39,40^. Although sibling species rarely interbreed, hybrids of *An. coluzzii* and *An. gambiae s.s.* were found in low proportions, as observed in other studies in Ghana^10,41^. Insecticide resistance patterns varies with the species of the *An. gambiae* complex and gene expression profiles may vary from one species to the other^42,43^. A big limitation of our study was that differential gene expression profiles of metabolic and cuticular genes were not done according to species.

The over-expression of both metabolic and cuticular genes in resistant *An. gambiae* mosquitoes from the study sites indicates that multiple resistance mechanisms (metabolic and cuticular resistance) may be involved in insecticide resistance in *An. gambiae* s.l populations from Ghana. The gene expression profile of metabolic and cuticular genes varied across the study sites. Hence, there may be variations in the metabolic and cuticular genes involved in the development of insecticide resistance from one site to another. This highlights the need for further studies to determine the insecticide resistance status of local malaria vectors and the mechanisms involved to help in the development of targeted control strategies for malaria control and elimination in Ghana.

## Methods

### Study sites

The study was carried out in eight sites in the southern and northern parts of Ghana, from which larval collections were made during the rainy and dry seasons from June 2023 to September 2023. The sites were Opeibea (5°35′46.42″N, 0°11′01.43″W), Tuba (5°30’46” N, 0°23’20"W), Teshie (5°35’0"N 0°5’0"W), Nima (5°35’0"N 0°12’0"W), Kpalsogu (9°33′45.2″N, 1°01′54.6″W), Libga (9°35′32.26′′N, 0°50′48.8′′W), Tamale (9° 25’ 58.5084’’ N and 0° 50’ 54.4272’’ W) and Kulaa (9°26′59″N, 0°43′44″W) (Fig. 1). Opeibea, Tuba, Nima and Teshie are in the Greater Accra region in the southern part of Ghana. Opeibea and Tuba are urban irrigated agricultural sites with high herbicide, pesticide, and fertilizer use, creating abundance of mosquito breeding sites, potentially influencing insecticide resistance^11^. Teshie and Nima serve as controls, with poor drainage and stagnant water favoring *Anopheles* mosquito breeding, particularly during the rainy season.

Kpalsogu and Libga are rural communities in Northern Ghana. These sites are near irrigation dams, supporting year-round farming and mosquito breeding. Due to a high malaria burden in these sites, Kpasolgu and Libga were selected for annual IRS by the President’s Malaria Initiative (PMI) and Ghana National Malaria Control Programme (NMCP) since 2008, though IRS in Libga was discontinued after 2014^44^. Tamale and Kulaa are primarily residential and industrial areas with limited farming but also have mosquito breeding sites during the rainy season.

### Larval collection and rearing

*Anopheles* mosquito larvae were collected from their breeding habitats mainly ponds, dug out wells, swamps, drainage ditches, puddles within each of the study sites. *Anopheles* larvae sampled were transported to the insectary at the Department of Medical Microbiology, University of Ghana Medical School, Accra, where they were raised to adults under stable conditions (temperature: 25 ± 2 °C, 80 ± 4% relative humidity). The larvae were fed on TetraMin Baby fish food (Tetra Werke, Melle, Germany). Emerged adults were fed on a 10% sugar solution until use in WHO susceptibility bioassays or synergist bioassays.

### Insecticide susceptibility testing on adult mosquitoes

Susceptibility tests using WHO tubes were conducted according to the WHO protocol^45^ to determine phenotypic resistance. Two to 5-day-old female mosquitoes were exposed to papers impregnated with the pyrethroids permethrin (0.75%) and deltamethrin (0.05%), Alphacypermethrin (0.05%), the organophosphates pirimiphos-methyl (0.25%), malathion (5%) and the carbamate bendiocarb (0.1%). In each test, 120 mosquitoes were exposed to the insecticide-impregnated papers, and oil-impregnated papers as controls. Each test comprised six replicates (four treatments and two controls). Mosquitoes were exposed for 1 h and the knockdown was recorded every 10 min during the 60-min exposure period. Mortality was recorded after a 24-h recovery period. Alive (resistant) and dead (susceptible) mosquitoes were separately stored in 1.5 ml Eppendorf tubes with silica gel and some kept in RNA later for subsequent molecular tests.

### Synergist assays with PBO

Piperonyl butoxide (PBO) synergist assay was performed to establish the role of cytochrome P450s in the development of resistance in the *Anopheles* mosquitoes. This synergist assay was performed according to WHO criteria^6^ using unfed females aged 2–5 days. Each test had four replicates of 20 female *Anopheles* mosquitoes, with each pre-exposed to 4% PBO impregnated test papers for one hour to suppress oxidase enzymes^6^. After pre-exposure to PBO, the mosquitoes were immediately exposed to each of the three pyrethroids (0.05% deltamethrin, 0.75% permethrin and 0.05% alphacypermethrin) separately for another hour. Two control tubes were run in parallel at any time of the testing. Knockdown was recorded during the 60 min period and mortality after 24 h.

### Morphological and molecular identification of *Anopheles* mosquitoes

Mosquitoes used for the susceptibility tests were morphologically identified using identification keys by Gillies and Coetzee^46^. A subset of 400 *An. gambiae* mosquitoes used for the susceptibility tests were further distinguished molecularly using PCR and RFLP-PCR. The legs of each mosquito were used for DNA extraction. Four sets of primers (*Anopheles gambiae*, *An. arabiensis*, *An. melas*, and universal primer) were used in PCR for the identification of members of the *An. gambiae* s.l. species complex. *Anopheles gambiae* s.l were distinguished into their sibling species (*Anopheles gambiae* s.s. and *An. coluzzii*) by PCR-RFLP using the method of Fanello et al.^47^.

### RNA extraction and cDNA synthesis for metabolic and cuticular resistance determination

Pyrethroid resistant and susceptible *An. gambiae* s.l from the susceptibility tests for each site and the *KISUMU* susceptible lab strain were used to investigate the role of metabolic and cuticular genes in resistance. The mosquitoes were stored in RNA later at − 20 °C and grouped in pools of 10 in 1.5-ml Eppendorf tubes. Total RNA was extracted from three pools of mosquitoes (pyrethroid resistant, pyrethroid susceptible and unexposed groups) using the ZYMO *Quick-RNA™ Miniprep Kit* following the manufacturer’s protocol. The cDNA was synthesized using Protoscript II First Strand cDNA Synthesis kit, New England Biolabs (United Kingdom) according to the manufacturer’s protocol. The total RNA and synthesized cDNA were stored at − 80ºC.

### Quantitative RT-PCR for metabolic and cuticular gene products

The study analyzed four Cytochrome P450 genes (*CYP6P3*, *CYP6M2*, *CYP9K1*  and *CYP4G16*) because of their importance in detoxification, one GST gene (*GSTE2*) and three Cuticular genes (*CLPCG*3, *CPR124*, *CPR129*) using well-described protocols^14,24,26,48^. Quantitative RT-PCR was performed using the Bio-Rad RT-PCR machine. Reactions were carried out in a final volume of 10 µl consisting of 5 µl SYBR Green Master Mix (Roche, Indianapolis, IN), 10 µM of each primer and 2.0 µl of cDNA. The qPCR assay was performed on the Bio-Rad Opus 96 PCR System (Bio-Rad) with an initial denaturation at 95 °C for 10 min, followed by 40 cycles of 95 °C for 10 s, 60 °C for 10 s). Standard curves for all primer sets were carried out using Kisumu cDNA as a reference.

### Statistical analysis

Statistical analysis was done in Statistical Package for Social Sciences (SPSS version 26) to compare bioassay mortalities for each insecticide among study sites. Homogeneity tests of percentages and averages were performed using standard chi-square tests with a 5% significance level threshold. The resistance or susceptibility status of the tested mosquito populations was evaluated following WHO criteria^45^. The CT values obtained were used in determining the expression levels of the selected genes using the delta CT method^49^.The house keeping gene S7 (VectorBase: AGAP010592) was used as an internal control. The fold expression of the genes was calculated using the formula, Fold change = 2 −∆∆CT with normalization against the ribosomal protein S7.

## Electronic supplementary material

Below is the link to the electronic supplementary material.

Supplementary Material 1

## Data Availability

The datasets generated and/or analysed during this study are available from the corresponding authors on reasonable request.
